# Identification and Risk Assessment for Worldwide Invasion and Spread of *Tuta absoluta* with a Focus on Sub-Saharan Africa: Implications for Phytosanitary Measures and Management

**DOI:** 10.1371/journal.pone.0135283

**Published:** 2015-08-07

**Authors:** Henri E. Z. Tonnang, Samira F. Mohamed, Fathiya Khamis, Sunday Ekesi

**Affiliations:** 1 International Centre of Insect Physiology and Ecology (*icipe*), Nairobi, Kenya; 2 International Maize and Wheat Improvement Center (CIMMYT), Nairobi, Kenya; Federal University of Viçosa, BRAZIL

## Abstract

To support management decisions, molecular characterization of data and geo-reference of incidence records of *Tuta absoluta* (Meyrick) (Lepidoptera: Gelechiidae) were combined with data on the biology and ecology of the pest to estimate its climatic suitability and potential spread at regional and global scale. A CLIMEX model was developed and used for the global prediction of current and future climate-induced changes in the distributional shifts of *T*. *absoluta*. Results revealed that temperature and moisture characterized *T*. *absoluta* population growth while the pest ability to survive the cold, hot, wet and dry stress conditions are the primary characteristics defining its range frontiers. Simulated irrigation also played an important role in the model optimization. Model predictions suggest that *T*. *absoluta* represents an important threat to Africa, Asia, Australia, Northern Europe, New Zealand, Russian Federation and the United States of America (USA). Under climate change context, future predictions on distribution of *T*. *absoluta* indicated that the invasive nature of this pest will result in significant crop losses in certain locations whereas some parts of Africa may witness diminution in ranges. The following scenarios may occur: 1) *T*. *absoluta* damage potential may upsurge moderately in areas of Africa where the pest currently exists; 2) a range diminution in temperate to Sahel region with moderate upsurge in damage potential; 3) a range expansion in tropical Africa with reasonable upsurge of damage potential. These possible outcomes could be explained by the fact that the continent is already warm, with the average temperature in majority of localities near the threshold temperatures for optimal development and survival of *T*. *absoluta*. Outputs from this study should be useful in helping decision-makers in their assessment of site-specific risks of invasion and spread of *T*. *absoluta* with a view to developing appropriate surveillance, phytosanitary measures and management strategies.

## Introduction

Tomato leaf miner, *Tuta absoluta* (Meyrick) (Lepidoptera: Gelechiidae), is native to South America [1] and has been recorded from Argentina, Bolivia, Brazil, Chile, Colombia, Ecuador, Paraguay, Peru, Uruguay and Venezuela. The pest is predominantly found in areas below 1000 m above sea level. It is a devastating pest of tomato, *Solanum lycopersicum* L. (Solanaceae). Additionally the pest has also been found to attack other cultivated and wild solanaceae, such as pepper, *Capsicum annuum* L.; eggplant, *Solanum melongena* L.; tobacco, *Nicotiana tabacum* L.; potato, *Solanum tuberosum* L. and black nightshade, *Solanum nigrum* [2]. Recently, the pest was reported to expand its host range to garden bean, *Phaseolus vulgaris* L. (Fabaceae) [2]. Outside its native areas of distribution the pest has been reported for the first time in Spain in 2006, from where it is believed to have spread to other European countries including Italy (2008), France (2008), Albania (2009), Bulgaria (2009), Portugal (2009), Netherlands (2009), United Kingdom (2009) and Serbia (2011). In the Middle East region, it is known to occur in Israel (2010), Iran (2010), and Turkey (2010) [3–8]. It was later reported in the Canary Islands in 2012 [9]. In northern Africa, *T*. *absoluta* was found in Algeria (2008), Morroco (2008), Egypt (2009), Libya (2009), and Tunisia (2009) [1], [3], [10]. In 2012, Ethiopia, Niger, Senegal, and Sudan, were the countries in sub-Saharan Africa (SSA) region where *T*. *absoluta* was first detected [11–14]. Currently, the pest is found in Tanzania, Uganda and Kenya (S. A. Mohamed et al., unpublished data). Detections in Kenya were confirmed by using both morphological character states and molecular tool for species delineation (DNA barcoding). The fast spread of the pest across Africa may possibly be enhanced by trade, porous borders and fragile nature of the phytosanitary infrastructure coupled with inadequate implementation of quarantine measures.

Modeling of pest distribution is an important technique available to biologists and ecologists to understand and predict the past, current or future presence of species based on ecological observations and/or laboratory data. Theories so far developed for modeling species distribution can roughly be categorized in two main groups: the first class that considers climate (either directly or indirectly) as the major determining factor through its interactions with the biology of specific species [15–17], and the second class, takes into consideration multi-species interactions such as competition and predation as being the paramount component for the species presence in a region [16].

The present study is based on the first school of thought; which is characterized by two methodological approaches; namely mechanistic and correlative [18–19]. The mechanistic approach is constructed on the translation of environmental conditions into biologically relevant metrics (*e*.*g*. potential duration of activity) to capture environmental sensitivities of survivorship and fecundity for predicting species incidence in a region [18]. This modeling framework does not directly match climate; it rather models the species response to climatic factors and results into a geospatial metrics that indicates where the climate is suitable for the species development, survival and establishment [18]. The ‘correlative’ approach employs statistical or machine-learning algorithms to known occurrence records of the species with digital layers of environmental variables [19] for predictions. It uses datasets and simple functions to describe the species’ response to climatic factors [19]. Commonly, presence/absence data or occurrence data only, from different localities, are sufficient for generating risk maps. Such approaches project climate similarity (with no reference to biological response to climate) and the results indicate how well the climate in an area matches the climate where the species is located [20].

In assessing the potential risks of invasion and spread of *T*. *absoluta* in different countries of Africa, CLIMEX (Hearne Scientific Software Pty Ltd, Australia) was selected. This modeling platform combines both mechanistic and correlative approaches and provides distribution/information maps that use different spatial scales to envision the potential pest spread and dispersal. The objective of the modeling exercise in this context is to support strategic decision-making by mapping out where *T*. *absoluta* might potentially establish. Based on this hypothesis it is possible to provide global information where cultivated crops of the family Solanaceae may be subjected to damage by *T*. *absoluta*. Moreover, the study aims at identifying climatic factors, which determine the likelihood of success or failure of the development of *T*. *absoluta*. It is believed that the outcome of this study should be useful to better inform decisions on policy implementation and pest management practices such as pest surveillance and the implementation of appropriate phytosanitary measures to manage the spread of *T*. *absoluta* in Africa and beyond.

## Materials and Methods

### Ethics Statement

The study was not undertaken in national park or any protected areas. The crop tomato (*S*. *lycopersicon*) and the invasive insect pest (*T*. *absoluta*) involved in this study are not endangered or protected species.

### Molecular Identification of Samples from Kenya

Trap catches from Isiolo Kenya (00 19.536 N, 037 33.233 E, 1160 masl) were preserved in 95% ethanol for DNA extraction and voucher specimen from the same samples have been kept at *icipe* Arthropod Pathology Unit. DNA was extracted from the whole moth using the Isolate II Genomic DNA Kit (Bioline GmbH) as per manufacturer’s instructions. The extracted DNA was stored at– 20°C until required for amplification. PCR was carried out using universal primers, forward primer (LCO1490) 5’-GGTCAACAAATCATAAAGATATTGG-3’ and reverse primer (HCO2198) 5’-TAAACTTCAGGGTGACCAAAAAATCA-3’ [21] to amplify a 658 bp fragment of the *COI* gene. The PCR amplification was carried out in a 20 μl volume containing 1x reaction Buffer, 200 μm of dNTP mix, 0.4 pmol/μl of each primer, 2.5 mM, MgCl_2_, 1 unit *Taq* DNA polymerase (*MyTaq*, Bioline) and 1 ng DNA template. Standard cycling conditions of 5 min at 94°C, then 35 cycles of 30 s at 94°C, 1 min at annealing temperature of 45°C and 1 min at 72°C, followed by a final elongation step of 5 min at 72°C were used. The products were purified using Isolate II PCR and Gel purification kit (Bioline GmbH) according to manufacturer’s instructions and subsequently sequenced in both directions using ABI 3700 genetic analyzers. Sequences obtained were assembled and edited using Chromas version 2.13 (Technelysium Pty ltd, Queensland, Australia). Consensus sequences from both the forward and reverse strands were generated and were then queried through BLASTN in the GenBank database provided by the National Centre of Biotechnology Information (NCBI) (http://www.ncbi.nlm.nih.gov/) for identification purposes and to check for similarity with organisms already identified. Furthermore, additional sequences were obtained from GenBank and a multiple alignment was done using ClustalX version 2.1 [22]. A phylogenetic tree was constructed using MEGA version 6.0 [23] through the neighbor-joining method with a bootstrap test of 1000 replications [24] using Kimura 2-parameter (K2P) distance model [25].

### *Tuta absoluta* Occurrence Data and Geo-Referencing

A search for information related to occurrence/incidence of *T*. *absoluta* was carried out from June 2013 to December 2014 using Web of Science, Google, PubMed and MEDLINE. The keywords used during the search comprised of the following: *T*. *absoluta*, tomato leafminer; South American tomato pinworm; pepper (*C*. *annuum*; Solanaceae); eggplant (*S*. *melongena*; Solanaceae); tobacco (*N*. *tabacum*; Solanaceae); potato (*S*. *tuberosum*; Solanaceae), solanaceous weeds and garden bean (*P*. *vulgaris*; Fabales: Fabaceae).

Geo-referencing a locality was centered on assigning geographical coordinates to the recorded description of that locality. The coordinates were assigned to records by searching the name of the site in the databases using Google Earth v.6.1 (GOOGLE) and on the maps with ArcGIS v.9.2 (ESRI). The coordinates were measured in decimal degrees and WGS 1984 datum. The combination of the analysis from database related to spatial interpretation and the output represented as map data were organized in 8 fields: continent, country, location, latitude, longitude, citations, references and the status of the species with categories in conformity with the European and Mediterranean Plant Protection Organization (EPPO) standards [26]. This information was used to generate MS Excel (Microsoft Corporation) datasets with detailed descriptions of the point locations where *T*. *absoluta* has been reported. *Tuta absoluta* incidence data obtained from collaborators in Eretria, Niger, Sudan, and Tunisia were also included in the datasets. The geo-referenced datasets of *T*. *absoluta* occurrence were used to produce a map depicting the current distribution of *T*. *absoluta*.

### CLIMEX Modeling Platform

CLIMEX modeling theory is based on the species approximation responses to temperature, moisture and sometimes day-lengths and diapause [27]. The conceptual framework is entrenched on the assumption that knowledge on occurrence of species in a particular location can guide the inference of its tolerance to prevailing environmental conditions [16], which can further aid in predicting potential establishment range. CLIMEX attempts to mimic the mechanisms that limit species geographical distributions and determine their seasonal phenology and to a small magnitude their relative abundance [16–17], [27–28]. The model is parameterized with environmental observations and eco-physiological information of the species growth, development and survival [16–17]. CLIMEX platform uses a database of meteorological variables to derive weekly and annual indices [17]. The potential for population growth is described by a weekly growth index (GI_W_), which is combined to generate the annual growth index (GI_A_). Weekly stress indices (cold, hot, wet, dry and their interactions) are related to define the ability of a population to survive extreme circumstances [27]. Growth indices were inspired from the Sprengel–Liebig law of the minimum and Shelford’s law of tolerance [29–30] while stress indices are used to illustrate the observed patterns of species population response to extreme conditions [23]. The overall climatic suitability of a particular location for a given species is provided by an Eco-climatic Index (*EI*), which combines the annual potential for population growth (*GI*_*A*_), the stresses (*SI*) that limit species survival and the factors limiting interacting between these stresses (*SX*) [17], [31].

EI=GIA*SI*SX (1)

### Meteorological Databases

CLIMEX modeling platform inputs weekly data from the averages of the following variables: maximum and minimum temperatures, relative humidity and rainfall. The climate data are sourced from the global change community through the Climatic Research Unit (CRU) in Norwich UK, available at http://www.cru.uea.ac.uk/. This data bank is made of point location with records from about 3,000 meteorological stations worldwide as well as interpolated climatic grids [31–32]. Due to low-density weather stations for Africa in CLIMEX database, additional weather data for East Africa were extracted from the FAOCLIM agro-climatic database [33]. With the supplemented datasets inputted into CLIMEX, a higher spatial resolution (5 arc-minutes) database was produced and used for developing the potential climatic suitability of *T*. *absoluta* in East Africa.

### Scenarios of Irrigation and Climate Change

CLIMEX platform has features for analyzing the potential implications of irrigation and climate change on the seasonal phenology and survival of species as well as their future potential geographical distribution. When using the irrigation function, it treats all regions equally and only affects the amount of additional simulated rainfall [31]. In calibrating CLIMEX, a seasonal irrigation with a ‘top-up’ amount of water to increase the effective rainfall to 3.6 mm per day option was selected. For climate change analysis, a rise of 1.5°C Africa wide temperature and 10% increase in rainfall from March 2—September 30 and 10% decrease in rainfall for the rest of the year, were considered.

### Potential Distribution of *T*. *absoluta* using CLIMEX

The developed model was fitted using inductive and deductive procedures. Inductive approach guided in fitting climatic trauma functions that describe *T*. *absoluta* range boundaries and deductive procedure was applied to express parameter values based on experiments of the species feedbacks to climatic factors and phenological observations [7], [34–35]. Practically, the model parameters were repetitively adjusted and the function "*Compare location*", which describes the potential geographical distribution of species, as controlled by weather variables was subsequently run until the estimated potential *T*. *absoluta* range best coincided with the known distribution of South America [16]. Calibration of the population growth and survival parameters was manually and iteratively conducted. The exercise was guided by biological information such as developmental threshold temperatures and the species’ feedback to different levels of natural and adequate wetness parameters [7], [34–35].

### Population Growth Parameters

Eight (8) parameters were used to define conditions suitable for *T*. *absoluta* population growth. Lower temperature threshold (DV0) and upper temperature threshold (DV3) captured the temperature optima and bounds for growth. Lower soil moisture (SM0) and upper soil moisture threshold (SM3) represent ideal wetness and constraint for growth. Primary values for these parameters were sourced from published data on *T*. *absoluta* developmental characteristics [3], [34]. The final values of population growth were obtained when stress functions were able to explain a greater proportion of *EI*. Degree-days per generation (PPD) was used to approximate the potential number of generations *T*. *absoluta* can complete per year at a given location under defined conditions.

### Survival Parameters

After the calibration of population growth parameters, mortality variables were inductively included to narrow the predicted distribution of *T*. *absoluta* to concur with known distribution patterns of South America. Three parameters (lower developmental temperature threshold (DVCS), weekly temperature threshold cold stress (TTCS) and weekly rate of accumulation of cold stress (THCS) were applied to characterize mortality due to extreme cold that prevents the distribution of *T*. *absoluta* in certain regions of South America. Additionally, cold stress accumulation rate (DHCS) and weekly degree-day threshold for cold stress (DTCS) were applied complementarily for taking into account mortality resulting from prolonged periods of cold, which may temporally occur and constraint *T*. *absoluta* incidence in unfavorable season. Due to lack of data, weekly heat stress temperature threshold (TTHS) and heat stress accumulation rate (THHS), which are additional CLIMEX parameters for inferring mortality resulting from extreme heat that constraints the distribution of species were not used. To eliminate areas of South America where there is no incidence of *T*. *absoluta*, the wet stress soil moisture threshold (SMWS), weekly wet stress accumulation rate (HWS), dry soil moisture dry stress (SMDS) and weekly rate of accumulation of dry stress (HDS) pressure factors were calibrated.

### Model Evaluation

For testing the predictive ability of the developed model, a procedure using independent data; meaning records from different continents, not considered during model parameters estimation was adopted [16]. The parameters used for developing the *EI* potential distribution map for South America that correctly fit the geo-reference incidence datasets in this region were applied to predict the potential distribution of *T*. *absoluta* in Europe and Mediterranean basin. The obtained map in Europe and vicinity was then compared with the known incidence point locations in the region. Similar procedure was performed for Africa to generate the predictive *EI* map of *T*. *absoluta*, which was matched with geo-reference location points obtained from field surveys in particular regions of the continent. Additionally, a quantitative point-by-point geographical evaluation was conducted for different locations around the world (Brazil, Egypt, Turkey and Bulgaria) to estimate the potential number of generations per year that *T*. *absoluta* could complete at natural conditions. The selection of the above four locations was based on the available historical field and experimental data on the number of generations per year of the pest [36–39].

## Results

### *Tuta absoluta* Molecular Identification

**Fig 1:**
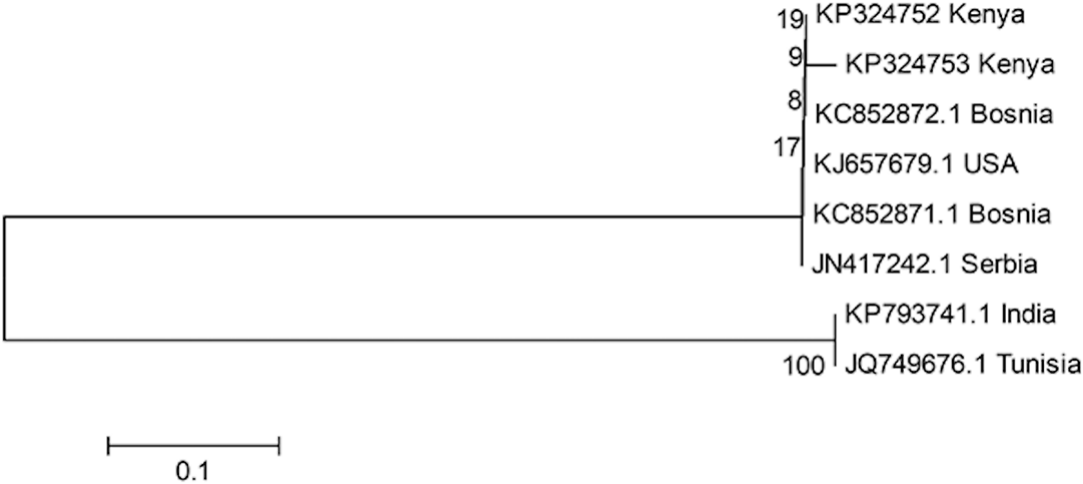
Phylogenetic tree of *T*. *absoluta* samples from Kenya and others obtained from GenBank.

Four (4) individuals were sequenced and the sequences were deposited at the GenBank with accession numbers KP324752 and KP324753. The Blast search through the NCBI BLASTN linked all the four individuals to *T*. *absoluta* sample from Serbia of accession number JN417242, with an E-value of 0 and with a probability of 99% match. This confirmed the identity of the moth as *T*. *absoluta*, an alien pest to Kenya. The optimal phylogenetic tree with the sum of branch length = 0.96778729 is shown in Fig 1. The analysis involved 8 nucleotide sequences. Codon positions included were 1st+2nd+3rd+Noncoding. All positions containing gaps and missing data were eliminated. There were a total of 579 positions in the final dataset. The Kenyan sample linked closely to the Bosnian, USA and Serbian samples while the sample from Tunisia and India occupied a different branch.

### *Tuta absoluta* Occurrence Data and Geo-Referencing

**Fig 2:**
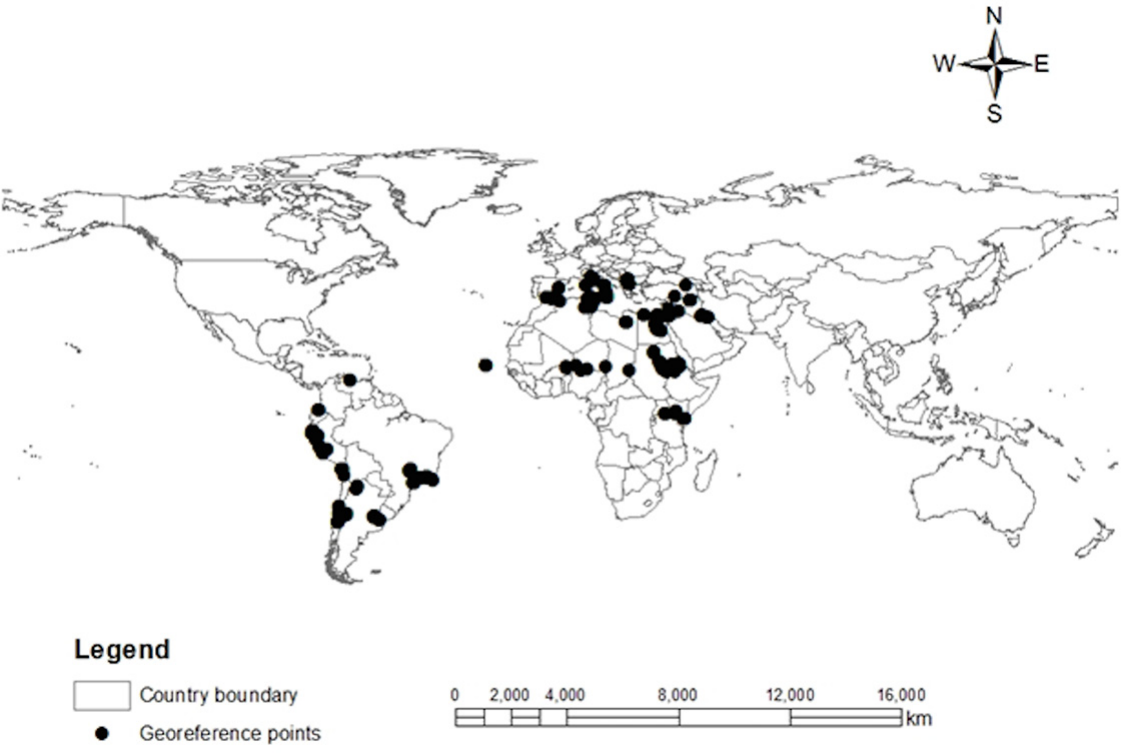
Global known geo-reference location points of *T*. *absoluta*. Point locations in South America correspond to its native distribution, and were obtained from the published literature. Point locations in Africa correspond to survey conducted by the authors of the article.

A total of 498 occurrence records of *T*. *absoluta* belonging to 4 continents were obtained from the literature search as follow: Africa (79), Asia (52), Europe (132) and South America (236). Fig 2 shows the current known geo-reference occurrence location points of *T*. *absoluta* in the world. Point locations of South America, which were used for model parameters estimation (Table 1), are made of records from Argentina (33), Brazil (74), Chile (63), Ecuador (2), Peru (71) and Venezuela (2).

**Table 1:** Parameter values used to build a potential distribution for *T*. *absoluta* using CLIMEX modeling platform.

Characteristic	Description—parameter	Value	Unit
	Lower temperature threshold (DV0)	8.0	°C
	Lower optimum temperature (DV1)	20	°C
	Upper optimum temperature (DV2)	30	°C
	Upper temperature threshold (DV3)	42	°C
**Population growth**	Lower soil moisture threshold (SM0)	0.1	SMC
	Lower optimum soil moisture (SM1)	0.4	SMC
	Upper optimum soil moisture (SM2)	1.5	SMC
	Upper of soil moisture (SM3)	4.0	SMC
	Degree-days per generation (PDD)	460	°C days
	Lower developmental temperature threshold (DVCS)	8.0	°C
	Weekly temperature threshold cold stress (TTCS)	3.0	°C
	Weekly rate of accumulation of cold stress (THCS)	-0.001	Week ^-1^
	Cold stress accumulation rate (DHCS)*	-0.0001	Week ^-1^
**Survival**	Weekly degree-day threshold for cold stress (DTCS)*	15	°C days
	Weekly heat stress temperature threshold (TTHS)	-	°C
	Heat stress accumulation rate (THHS)	-	Week ^-1^
	Weekly wet stress soil moisture threshold (SMWS)	2.0	SMC
	Wet stress accumulation rate (HWS)	0.002	Week ^-1^
	Soil moisture dry stress (SMDS)	0.1	°C
	Weekly rate of accumulation of dry stress (HDS)	-0.01	Week ^-1^

* Alternative cold stress hypothesis

### Population Growth Parameters

Table 1 contains the parameter values used to build the potential distribution of *T*. *absoluta*. DV0 was fixed to 8.0°C, as this was the minimum developmental threshold found in the literature [3], [40–41]. DV1 was fixed to 20°C and DV2 at 30°C. This range well encompasses the estimate of the optimum developmental threshold reported to be around the value of 25°C [37]. Many studies have reported the DV0 value for *T*. *absoluta* [41–42]; however, we have not encountered any study that showed the pest’s estimated value for DV3. Due to *T*. *absoluta* occurrence in cold temperate and warm tropical regions, we presumed that *T*. *absoluta* is highly adapted to wide range of temperatures, which offers the pest possibility to thrive under harsh conditions in Northern Sudan where daily temperatures constantly fluctuate and may occasionally reach up to 49°C in summer. Therefore, DV3 average weekly values was fixed to 42°C. *Tuta absoluta* may also be tolerant to dryness, requiring less water supply [3], [41], which may be the reason why it flourishes well in hot and dry areas. The lower moisture threshold was set to 0.1 and the optimum soil moisture at 4. PDD was allocated the value of 460-degree days as reported in literature [3], [41].

### Survival Parameters

The values of survival parameters (Table 1) were adjusted such that the developmental cold stress (CS) temperature threshold was fixed to 8°C [3], [41]. TTCS was fixed to 3, denoting that *T*. *absoluta* can tolerate up to 3 cold days a week before it succumbs to CS [3] related damage. As the frequency and duration of coldness upsurge, the probability of damage in pest tissues increases. THCS was fine-tuned until the coldest known location where the pest has been reported was captured as being hardly suitable for this species persistence. CS was assumed to start accumulating at a rate of -0.0001 for DHCS per week with DTCS of 15°C days. Due to lack of data, mortality due to extreme heat; THHS and TTHS were not considered. WS was estimated to accumulate at a rate of 0.002 (HWS) per week with a value of 2 for SMWS, equivalent to the level of water stock aptitude. The average SMDS was set to 0.1, signifying that *T*. *absoluta* is sensitive to dry stress.

### Potential Distribution Maps

CLIMEX model combines the growth and stress indices into an overall *EI*, ranging from 0 to 100 with the value of equal to 0 signifying unsuitable and *EI* of 100 representing optimal suitability for the year-round survival of a species [17]. The scaling for zones of suitability was made in accordance with the developed geo-reference map of *T*. *absoluta* incidence in South America. Four classes of *EI* were considered as shown in Fig 2: Class 1 (*EI* = (0–5)) indicates that the location is not suitable for *T*. *absoluta* survival. Class 2 (*EI* = (5–20)) represents zones with very moderate levels of suitability for the survival of *T*. *absoluta*. Class 3 (*EI* = (20–50)) indicates zones with high risk of establishment of *T*. *absoluta*. Class 4 (*EI* > 50) indicates the likelihood of long-term favorable zones that support the survival and establishment of *T*. *absoluta*.

**Fig 3:**
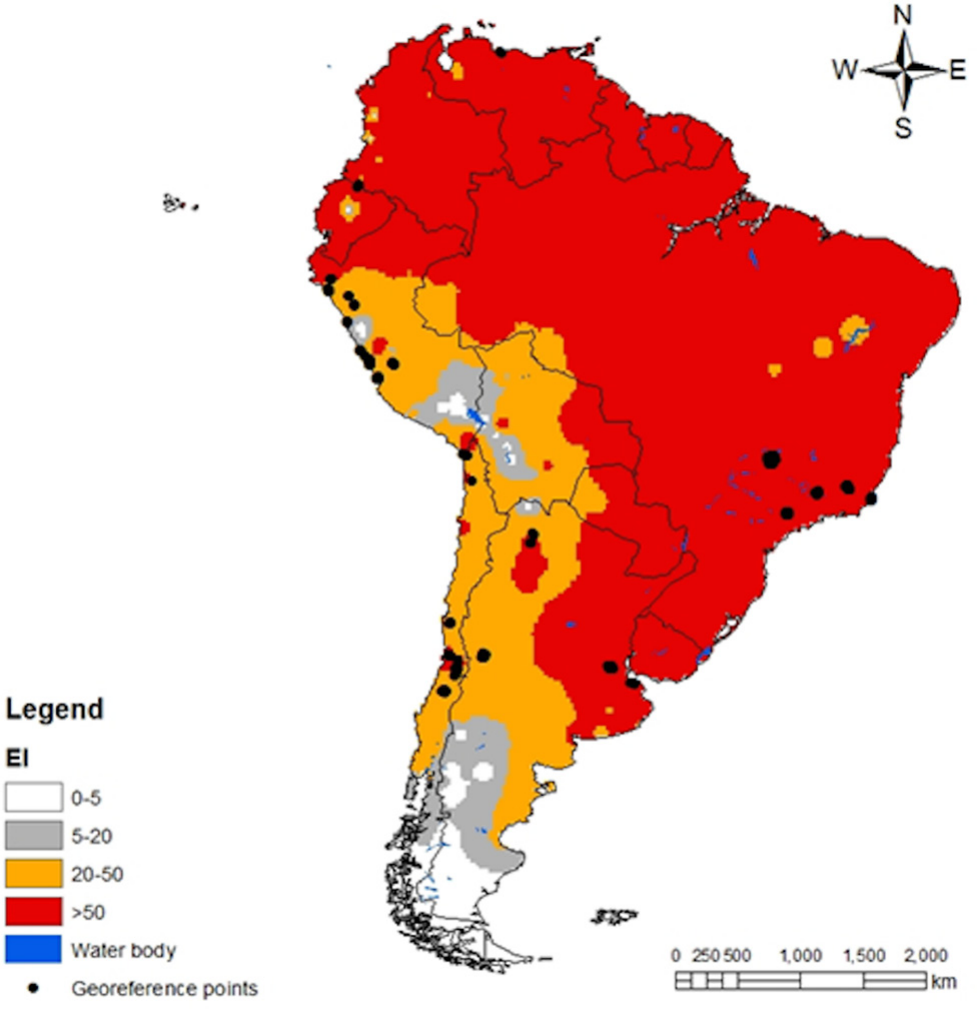
CLIMEX climatic suitability indices for *T*. *absoluta* in South America considered as the native region of the pest. Predictions are based on the eco-climatic index (EI), a measure of climatic suitability scaled from 1–100, for locations within CLIMEX’s platform station database. EI = (0–5) location is not suitable; EI = (5–20) moderate level of suitability; EI = (20–50) high risk of establishment and EI > 50 very high likelihood of long-term survival. The black dotes are the geo-reference points obtained from literatures.

**Fig 4:**
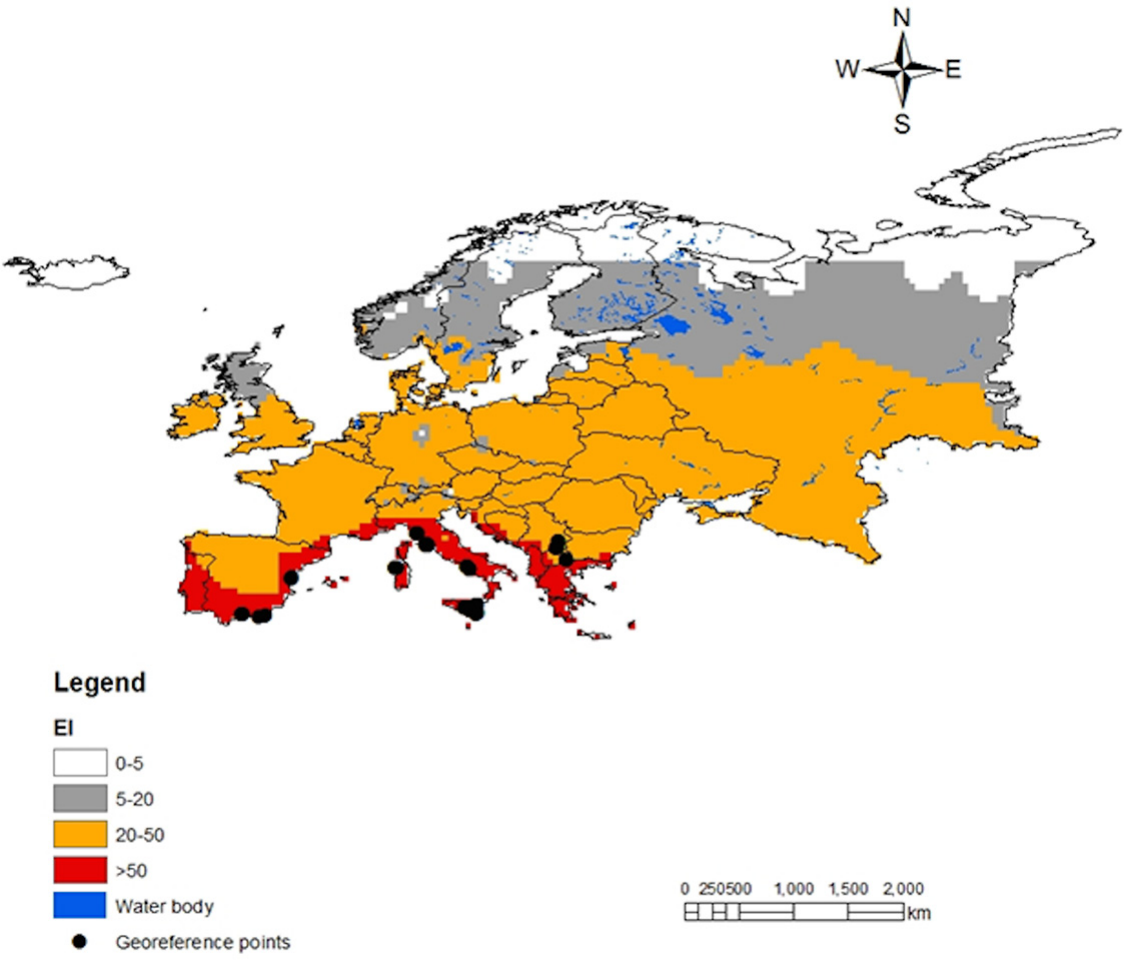
CLIMEX climatic suitability indices for *T*. *absoluta* in Europe. Predictionsare based on the eco-climatic index (EI), a measure of climatic suitability scaled from 1–100, for locations within CLIMEX’s platform station database. EI = (0–5) location is not suitable; EI = (5–20) moderate level of suitability; EI = (20–50) high risk of establishment and *EI* > 50 very high likelihood of long-term survival. The black dotes are the geo-reference points obtained from literatures.

**Fig 5:**
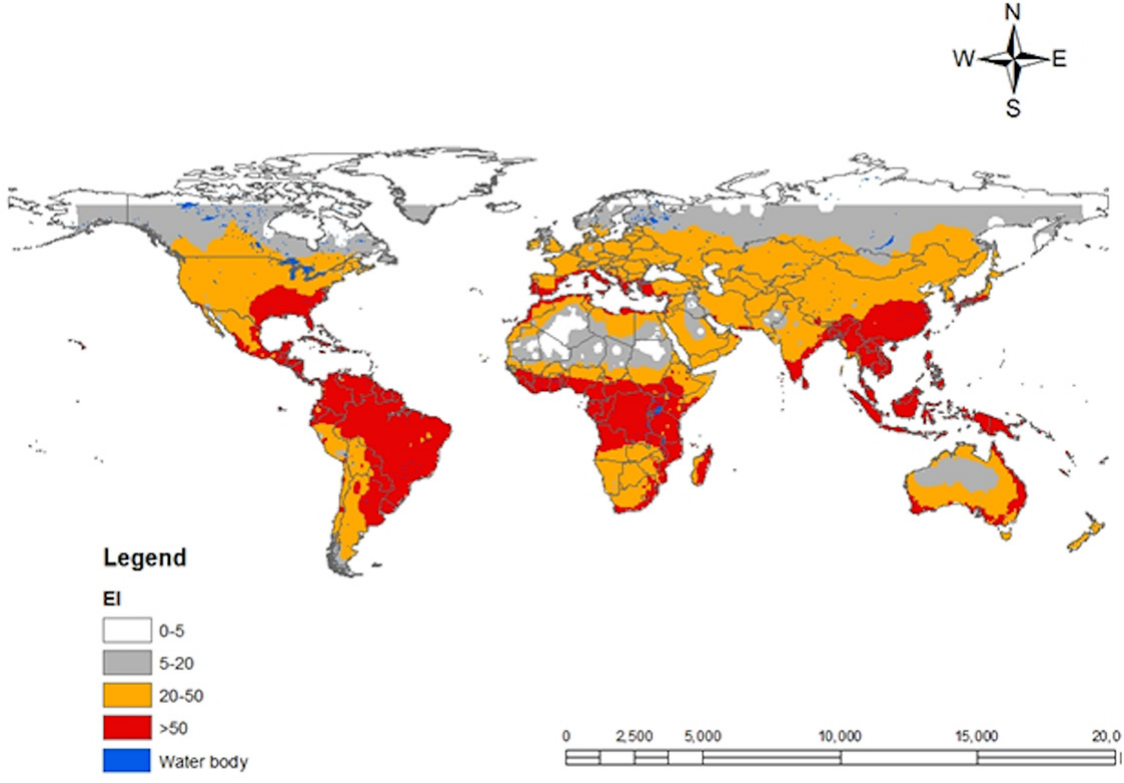
CLIMEX climatic suitability indices for *T*. *absoluta* in the world. Predictions are based on the eco-climatic index (*EI*), a measure of climatic suitability scaled from 1–100, for locations within CLIMEX’s station database. *EI* = (0–5) location is not suitable; *EI* = (5–20) moderate level of suitability; *EI* = (20–50) high risk of establishment and *EI* > 50 very high likelihood of long-term survival. The black dotes are the geo-reference points obtained from literatures.

Spatial predictions for South America locations within CLIMEX’s station database are shown in Fig 3. The predictions satisfactorily match the known distribution map for *T*. *absoluta* in the region and the geo-reference point locations belonging to class 3 and 4. Although the geo-reference records for Europe only include point location near the Mediterranean basin, *T*. *absoluta* potential distribution map in the region considers several countries with high risk for the pest invasion. By January 2015, *T*. *absoluta* has been reported in over 20 European countries [3], [39]; majority of which are in the southern, central and western parts of Europe. The present distribution map (Fig 4) showed that the northern countries (Belarus, Lithuania, Estonia, Denmark) might as well be at risk of *T*. *absoluta* invasion. A global projection (Fig 5) of the developed model indicated a high risk of invasion and establishment of *T*. *absoluta* in the world. From threshold developmental temperatures and degree-day accumulation, *T*. *absoluta* probably cannot survive winter conditions in the cold regions. However, transient populations may possibly survive in the field during the summer season and year round in greenhouses.

**Fig 6:**
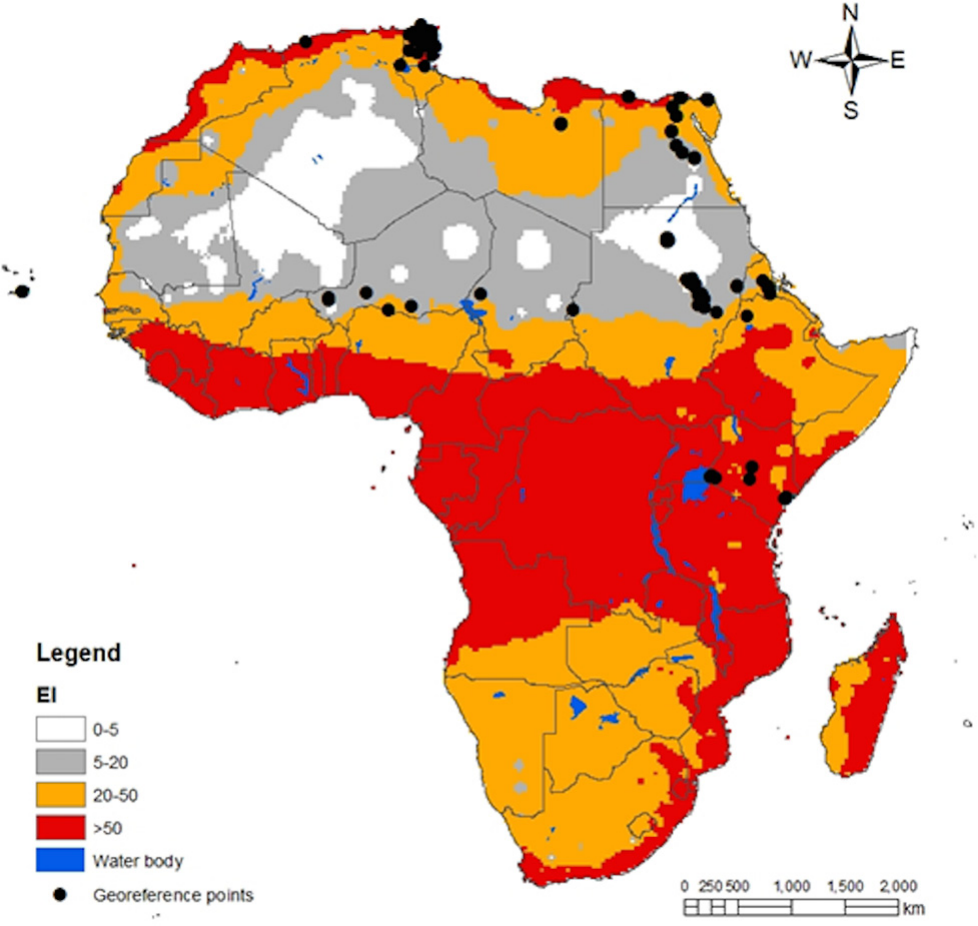
CLIMEX climatic suitability indices for *T*. *absoluta* at in Africa. Predictions are based on the eco-climatic index (*EI*), a measure of climatic suitability scaled from 1–100, for locations within CLIMEX’s station database. *EI* = (0–5) location is not suitable; *EI* = (5–20) moderate level of suitability; *EI* = (20–50) high risk of establishment and *EI* > 50 very high likelihood of long-term survival. The black dotes are the geo-reference location points obtained from surveys.

**Fig 7:**
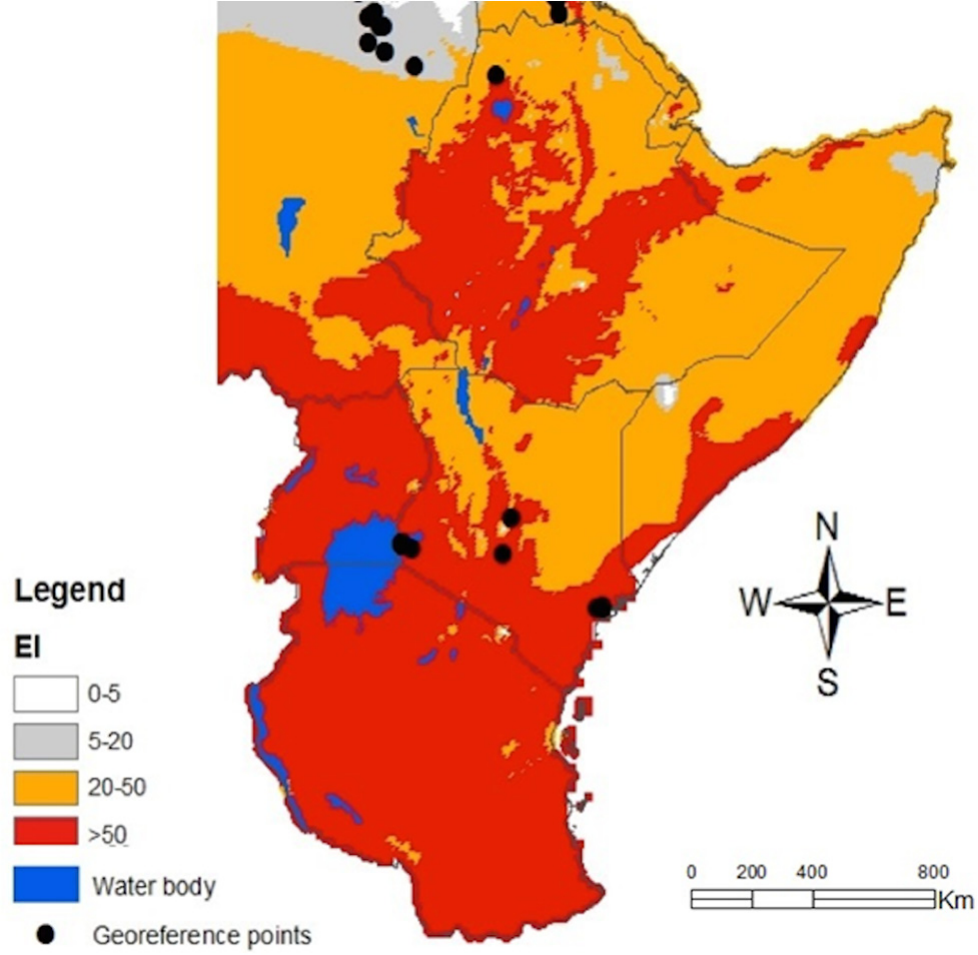
CLIMEX climatic suitability indices for *T*. *absoluta* in East Africa. Predictions are based on the eco-climatic index (*EI*), a measure of climatic suitability scaled from 1–100, for locations within CLIMEX’s station database. *EI* = (0–5) location is not suitable; *EI* = (5–20) moderate level of suitability; *EI* = (20–50) high risk of establishment and *EI* > 50 very high likelihood of long-term survival. The black dotes are the geo-reference points obtained from surveys.

*Tuta absoluta* has been reported in many countries in the Middle East (Fig 4) and in Africa (Fig 6). The model predictions of the pest incidence in Africa reasonably match the current known distribution of *T*. *absoluta*. Warmer climates (similar to South America) that prevail in these regions may lead to soaring damage of the crops by this pest. The supplement datasets at higher spatial resolution of 5 arc-minutes from East Africa provided more accuracy in predictions for East Africa (Fig 7) as compared to the predictions for Africa (Fig 6). Referring to the classification of *EI* above, East Africa region has high probability for the pest to invade and naturalize.

### *T*. *absoluta* Potential Number of Generations per Year

Published and predicted data on the number of generations’ that *T*. *absoluta* can produce per year is shown in Table 1. The model prediction in different locations yielded the following number of generations per year of *T*. *absoluta* in different countries and localities: Brazil, *Permambuco* 10.1; Egypt, *El-Behaira* 11.6, *Giza* 12.3, *Fayoum* 11.4 and *Qena* 13.4 generations per year; Turky, *Sanliurfa* 4.9; Bulgaria, *Pleven* 4.8, *Plovdiv* 5.1, *Sliven* 4.9, *Sofiya* 3.9 and *Varna* 4.9. Empirical results [36–39] at these locations revealed similar number of generations per year as shown in Table 2.

**Table 2:** Number of *T*. *absoluta* generations produced per year in different locations around the world. Comparison of the developed model outputs using CLIMEX and experimental results published in the literatures.

Locality/Country	Number of generation per year		References
	Model	Experimental	
Pernambuco (Brazil)	10.1	9	Vivan et al. 2002
El-Behaira (Egypt)	11.6	11	Abolmaaty et al (2010)
Giza (Egypt)	12.3	12	Abolmaaty et al (2010)
Fayoum (Egypt)	11.4	12	Abolmaaty et al (2010)
Qena (Egypt)	13.4	13	Abolmaaty et al (2010)
Sanliurfa (Turky)	4.9	4	Mamay and Yanik (2012)
Pleven (Bulgaria)	4.8	2–5	Karadjova et al. (2013)
Plovdiv (Bulgaria)	5.1	2–5	Karadjova et al. (2013)
Sliven (Bulgaria)	4.9	2–5	Karadjova et al. (2013)
Sofiya (Bulgaria)	3.9	2–5	Karadjova et al. (2013)
Varna (Bulgaria)	4.9	2–5	Karadjova et al. (2013)

### Potential Irrigation and Climate Change Induced Shift for *T*. *absoluta* Distribution in Africa

Topping up the precipitation via irrigation to 3.6 mm per day in the growing season amplifies the climatic suitability for *T*. *absoluta* in several regions such as the African Sahel and therefore improves the model predictions. For example, *EI* values of dry regions like Niger (*Maradi*, *Ouallam*, *Tohoua* and *Zinder*) and Sudan (*Blue Nile*, *Elabas*, *Gazira*, *Kassala*, *Khartoum*) with no or low level of suitability become probable habitat for *T*. *absoluta*. Furthermore, these localities have recorded severe damage by the pest, proving their suitability as habitats of *T*. *absoluta*, despite their low *EI* values. *Tuta absoluta* would possibly show a wide annual population density fluctuation in these localities [17].

**Fig 8:**
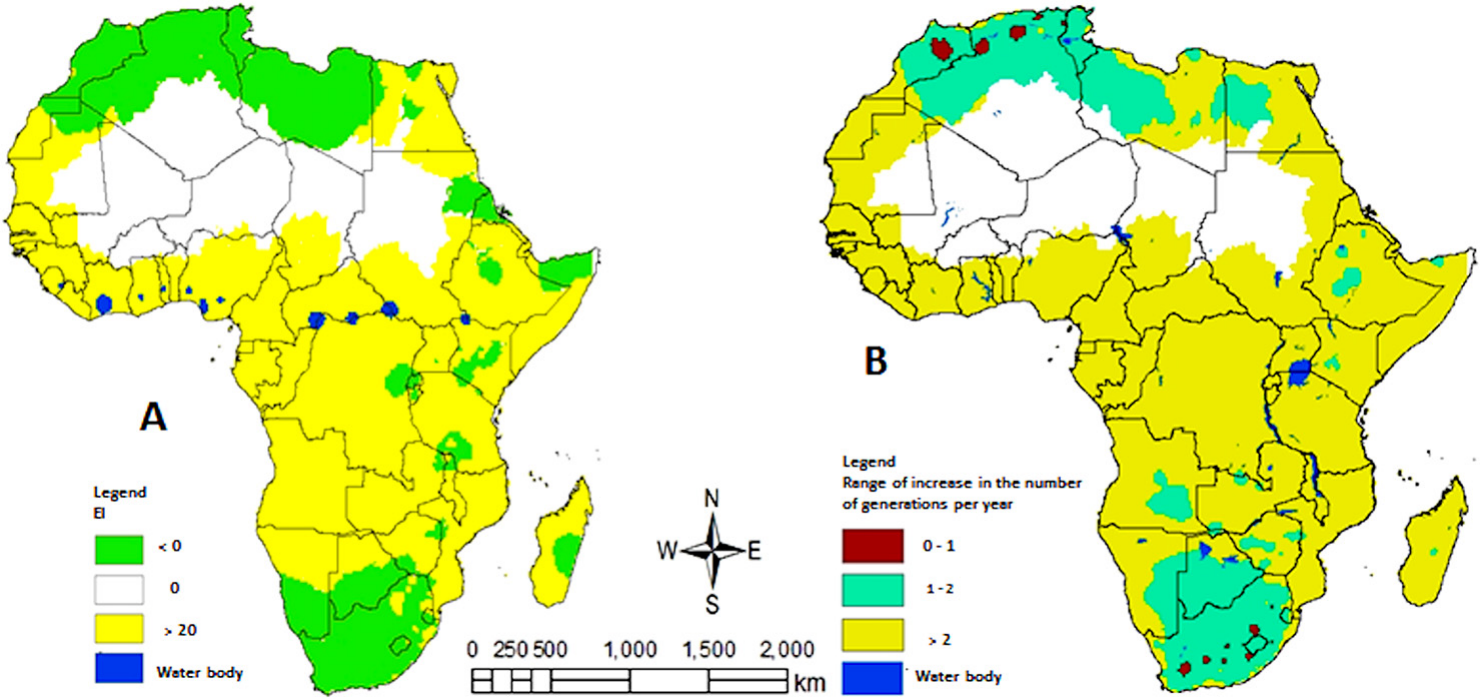
(A) Potential range shifts in the distribution of *T*. *absoluta* in Africa using the eco-climatic indices *EI* under climate change scenario (a rise of 1.5°C Africa wide temperature and 10% increase of rainfall from March 2—September 30 and 10% decrease in the rest of the year). The map was produced from the difference between the values of *EI* of the predicted future *T*. *absoluta* distribution (obtained when applying climate change criteria) and the distribution of the pest originated from current climate (year 2000) in Africa. *EI* = 0 demonstrates no range shift; *EI* < 0 signifies a reduction of climatic suitability *EI > 0* represents an increase in the likelihood of survival and permanent establishment of the species. (B) Potential range of increase in number of generations per year of *T*. *absoluta* under the selected climate change scenario.

The map in Fig 8(A) shows the difference between the values of *EI* from predicted future distribution of *T*. *absoluta* (obtained when applying climate change criteria) and the distribution of the pest originated from the model inputs with climate datasets of the year 2000 in Africa. A value of *EI* = 0 demonstrates no range shift. A negative value of *EI* signifies a reduction of climatic suitability for *T*. *absoluta* to persist in a region, whereas a positive *EI* represents an upsurge in the probability of survival and permanent establishment of the pest. Based on the total number of degree-days above the lower threshold temperature for *T*. *absoluta* growth, 1 to 2.5 generations were estimated as the potential value of increase in number of generations under the selected climate change scenario (Fig 8(B)). The temperate zones (southern and northern) of Africa may, with time, reduce in level of climatic suitability for *T*. *absoluta* establishment while significant area of the continent (western, central and eastern) will be more favorable for its establishment consequently contributing to long-term survival of *T*. *absoluta* in the continent.

## Discussion

### Molecular Identification

A pre-requisite to any management strategies is proper identification of the pest species. Molecular tools have been utilized for species identification due to limitations of morphological features [44]. The current molecular tool of choice is DNA Barcoding, which is a system that employs sequence diversity in short, standardized gene regions aiding in identification of species [45]. This standardized method for identifications of species focuses sequencing efforts on one target gene, cytochrome c oxidase subunit I (COI) [44–46]. DNA barcoding successfully identified the moth’s sample as *T*. *absoluta*. The pest is most likely on a downward incursion from North Africa towards SSA.

### Geo-Reference Information of *T*. *absoluta*

The information used to generate the worldwide distribution of *T*. *absoluta* contains numerous challenges; for instance, details of some records were given only at national and regional level making them unusable for geo-referencing exercise. Nevertheless, the produced *T*. *absoluta* geo-reference incidence and establishment map showed that the pest may be restricted to the 45° north and south latitudes of the temperate zones, which are above the borders of subtropical regions. However, the pest has been found without detailed knowledge of permanent establishment in certain countries marginally above this range (Ukraine, Russia [43] and United Kingdom [47]).

### CLIMEX Model Match with *Tuta absoluta* Native and Invaded Distribution

Among the various procedures and tools, accessible for pest risk assessment [48–50], CLIMEX was favored because of its track record of being able to model the potential distribution of invasive species with some level of reliability and accuracy [51–54]. CLIMEX platform offers an easy option for its outputs to be directly exported to ArcGIS v.9.2 (ESRI) for standardized spatial analysis. The representation of the outputs became practical, showing *T*. *absoluta* distribution ranges, which facilitate the analysis of results for explicit conclusions [16]. Another advantage of using CLIMEX is the ability of the tool to provide useful predictions with minimum input information. This was demonstrated by using only 230 geo-reference records for the whole of South America to develop and fine-tune the model of *T*. *absoluta* that offers acceptable results globally. In addition, it is appropriate that a model of species prediction at continental level is verified using independent incidence data sets of the same species from different continents [16], [31]. CLIMEX offers features to easily conduct such evaluation; thus the value of parameters produced to predict *T*. *absoluta* incidences in South America were reused to project its potential invasion and establishment in Europe and other parts of the world.

*Tuta absoluta* is reported to be a multi-voltine species with a high reproductive potential [3], [42]. Larvae do not undergo diapause when food and suitable ecological conditions are available; the species can produce up to 12 generations per year [42]. The high reproductive capacity contributes to the invasive nature of *T*. *absoluta*, which offers plausible justification of the assessment of its potential for establishment and spread. This article complements previous efforts, which were only limited to Europe and the Mediterranean basin, of calibrating CLIMEX mechanistic platform [3], [39]. A major advantage in the present model is the formulation of new hypotheses, including irrigation as a potential vehicle for the spread and permanent establishment of *T*. *absoluta* in other areas. This factor that was not considered in previous studies [3], [39], was very useful in this study as it affects both the growth and survival of *T*. *absoluta* by enabling populations to persist and florish in season and region with suboptimal climatic conditions. In warm regions such as North Sudan, crops are predominantly under irrigated cultivation, which may have contributed to the successful propagation and potential establishment of the pest in this country. In addition, *T*. *absoluta* may be able to establish permanent populations inside greenhouses with controlled temperatures, if irrigated host plants are available throughout the year; consequently, providing adequate justification for the presence of the pest in some cold countries of Europe [3–8]. Simulation of irrigation considerably increased the extent to which *T*. *absoluta* could invade areas with little rainfall and where tomato fruits and Solanaceous plants are cultivated in Africa, Asia and Australia. By including irrigation module into our model, it enhanced the level of suitability of the Sahel, an important region of tomato production that is witnessing a wider range of incidence for *T*. *absoluta*. With this module, even the marginally suitable habitats in parts of the Middle East and Australia also became potential habitats for invasion and potential establishment of *T*. *absoluta*.

The present model further considers that *T*. *absoluta* might tolerate higher moisture, threshold temperatures and temperature summation needed for development. These assumptions make the potential distribution patterns of the species more accurate in capturing incidence data from South America. Projections in Europe and other areas suggested that countries located at approximately 60° north (Denmark, Estonia, Latvia, Norway, Sweden, sections of United Kingdom and the Russian Federation) are also at risk of invasion by *T*. *absoluta*. Such findings are in agreement with the literature that mentioned erratic outbreaks of *T*. *absoluta* in some of these countries [3], [8], [43–47]. Regional trade of agricultural products may be playing an important role in the dispersal of *T*. *absoluta*. It has also been reported that the adult pest is capable of flying between farms [3], [47] during summer and potentially finding other suitable plants such as garden Solanaceae as alternative hosts [3], [47]. This further explains the spontaneous occurrence of *T*. *absoluta* in some of the cultivated plants in greenhouses and open fields in northern Europe.

Globally, the potential of invasion by *T*. *absoluta* is very high: Asia, Middle East, New Zealand, United Stated of America and a large section of Australia present high areas of suitability, persistence and spread of the pest. It is suggested that cold winters and lack of soil moisture may be critical factors determining the survival of *T*. *absoluta* in a region. Dry stress limits the climatic suitability in low precipitation zones and, likely, cold stress prevented the permanent establishment of the pest in the following regions: northern parts of Europe; the Russian Federation and Canada. Records of *T*. *absoluta* occurrence in hot and dry areas like *Zinder* (Niger) and *Khartoum* (Sudan) in Africa suggest that this pest is extremely heat-tolerant. It could survive in areas with little annual rainfall as long as host plants are available.

### *Tuta absoluta* as a Threat to Africa: Zooming to the Horn of the Continent

Surveys conducted in different parts of Africa showed that *T*. *absoluta* is considerably spreading very fast across the continent. The pest is currently reported in Algeria, Canary Island, Eritrea, Ethiopia, Egypt, Libya, Morocco, Niger, Senegal, Sudan [1], [3], [9–14] and in South Sudan, Kenya, Tanzania and Uganda (S.A. Mohamed et al., unpublished data). There is a high probability that *T*. *absoluta* is already in Somalia where it transited from Ethiopia and invaded Kenya. The potential distribution of this pest in Africa as observed from the developed model showed that it can invade and establish in most areas of the continent. This rapid spread may be attributed to the intensive cultivation and cross border trade of tomato fruits, which is the primary host of *T*. *absoluta*. In addition, the ecological and climatic conditions of Africa are similar to those of South America countries, the native region of the pest. According to published data, adult *T*. *absoluta* were found more than 6 miles (10 kilometers) away from tomato fields, suggesting that they can move long distances to colonize new areas by flying or drifting with the wind currents [3].

CLIMEX climate database only contains 720 stations for the whole Africa. Around the Sahara desert region, weather data is very scanty, probably justifying the failure of predictions to capture some localities where *T*. *absoluta* has been recorded. Due to such misrepresentation, supplementary datasets were added to generate a high-resolution predictive map for East African region. Zooming in, at countries of the horn of Africa showed that this region has a very high risk of invasion and permanent establishment of the pest. *Tuta absoluta* therefore, represents a significant threat to many horticultural crops considered as cash products in the region. The abundance of smallholder farmers may as well facilitate its rapid spread. Exercising vigilant phytosanitary inspections of imported plant germplasms and putting in place strict quarantine measures on exchange of planting materials and fresh harvest from endemic to non-endemic countries are recommended to curb the spread of this devastating pest.

### *Tuta absoluta* Potential Number of Generation per Year

The model predictions for the potential establishment of *T*. *absoluta* were validated with known geo-referenced distribution data. However, it was found convenient to further evaluate the predictive ability of the model by estimating the potential number of generations that *T*. *absoluta* may develop in different localities of the world. The outcome of this second validation technique was based on the findings from the literatures [36–37]. Hence increasing the expectation of the predictive ability of the model developed for *T*. *absoluta*.

### Change in Climate-Induced Distribution of *Tuta absoluta* in Africa

The International Panel for Climate Change (IPCC) has made a number of predictions for future climate change [55]. A warming of about 0.2°C per decade projected for the next two decades may possibly drive the expected range shifts of insects alongside the emergence of new pests in natural ecosystems which may change in response to altered temperature and precipitation profiles [56]. Africa is assumed to be the most vulnerable continent to climate change [55]; thus there is an urgent need to analyze possible impacts of climate change-induced changes on *T*. *absoluta* in this region. The present analysis and predictions on the future distribution of *T*. *absoluta* indicated that the invasiveness of this pest would be intensified and the number of the pest generation per year will increase, resulting in high yield losses in some locations. Depending on climatic zones (temperate, tropical, arid and semi-arid), the following possible scenarios may concurrently or individually occur: i) *Tuta absoluta* damage potential may progressively upsurge in regions of Africa where the pest already occurs; ii) a range diminution in temperate and semi-arid regions with moderate increase in damage potential; and iii) a range extension in tropical Africa with rationally high increase of the pest damage potential. It was reported [57] that the effects caused by global warming, precisely, temperature change on the metabolism of insects, are non-linear and may accelerate in warmer regions with little increase in temperature [57]. Such findings may be used to substantiate why future damage potential of *T*. *absoluta* is expected to rise in tropical than temperate Africa as predicted by the model. Another justification may be due to the fact that the continent is considerably warm, with the average temperature in many locations already near the threshold temperatures for optimum development and survival of the pest.
