# Correction: Identification and Risk Assessment for Worldwide Invasion and Spread of *Tuta absoluta* with a Focus on Sub-Saharan Africa: Implications for Phytosanitary Measures and Management

**DOI:** 10.1371/journal.pone.0138319

**Published:** 2015-09-11

**Authors:** Henri E. Z. Tonnang, Samira A. Mohamed, Fathiya Khamis, Sunday Ekesi

The second author’s name is spelled incorrectly. The correct name is: Samira A. Mohamed.

The affiliation for the second, third, and fourth author are incorrect. Samira A. Mohamed, Fathiya Khamis, and Sunday Ekesi are not affiliated with #2 but with #1 International Centre of Insect Physiology and Ecology (icipe), Nairobi, Kenya.
